# Synthesis, crystal structure and Hirshfeld surface analysis of naphthalene-2,3-diyl bis­(3-benz­yl­oxy)benzoate

**DOI:** 10.1107/S2056989023005571

**Published:** 2023-07-04

**Authors:** H. Anil Kumar, S. Selvanandan, H. T. Srinivasa, G. Venkateshappa, B. S. Palakshamurthy

**Affiliations:** a Department of Physics, Government First Grade College, Magadi, Karnataka 562120, India; b Department of Physics, ACS College of Engineering, Bangalore, Karnataka 560074, India; c Raman Research Institute, C.V. Raman Avenue, Sadashivanagar, Bangalore, Karnataka, India; d Department of Chemistry, UCS, Tumkur University, BH Road, Tumkur, Karnataka 572103, India; e Department of PG Studies and Research in Physics, Albert Einstein Block, UCS, Tumkur University, Tumkur, Karnataka 572103, India; University of Aberdeen, United Kingdom

**Keywords:** crystal structure, naphthalene, Hirshfeld surface, benzoate

## Abstract

In the title compound, C_38_H_28_O_6_, the dihedral angles between the naphthalene ring system and its pendant benz­yloxy rings *A* and *B* are 88.05 (7) and 80.84 (7)°, respectively. The dihedral angles between the *A* and *B* rings and their attached phenyl rings are 49.15 (8) and 80.78 (8)°, respectively. In the extended structure, the mol­ecules are linked by weak C—H⋯O and C—H⋯π hydrogen bonds, and π–π stacking inter­actions, which variously generate *C*(11) chains and *R*_2_^2^(12) loops as part of a three-dimensional network. The Hirshfeld surface [fingerprint contributions = H⋯H (42.3%), C⋯H/H⋯C (40.3%) and O⋯H/H⋯O (15.7%)] and inter­molecular inter­action energies are reported, with dispersion (*E*_dis_ = −428.6 kJ mol^−1^) being the major contributor.

## Abstract 2

In the title compound, C_38_H_28_O_6_, the dihedral angles between the naphthalene ring system and its pendant benz­yloxy rings *A* and *B* are 88.05 (7) and 80.84 (7)°, respectively. The dihedral angles between the *A* and *B* rings and their attached phenyl rings are 49.15 (8) and 80.78 (8)°, respectively. In the extended structure, the mol­ecules are linked by weak C—H⋯O and C—H⋯π bonds and π–π stacking inter­actions, which variously generate *C*(11) chains and 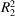 (12) loops as part of a three-dimensional network. The Hirshfeld surface [fingerprint contributions = H⋯H (42.3%), C⋯H/H⋯C (40.3%) and O⋯H/H⋯O (15.7%)] and inter­molecular inter­action energies are reported, with dispersion, *E*_dis_ at −428.6 kJ mol^−1^ being the major contributor.

## 1. Chemical context

Naphthalene, biphenyl or benzene rings can act as rigid cores in liquid crystal mol­ecules. A variety of banana-shaped, bow-shaped or bent-core ferroelectric liquid crystals were developed by incorporating a benzene ring as a rigid core (Noiri *et al.*, 1996 ▸; Srinivasa *et al.*, 2017 ▸). These types of compounds form lamellar and/or columnar mesophases (Szydlowska *et al.*, 2003 ▸) and they have been subjected to experimental and theoretical studies (Reddy *et al.*, 2006 ▸; Vaupotič, 2006 ▸). Liquid crystalline materials with a bent-core mol­ecule are attractive because they exhibit good physical properties and possess two-dimensional smectic phases that display qualitatively different physical properties than calamatic ferroelectric liquid crystals. 
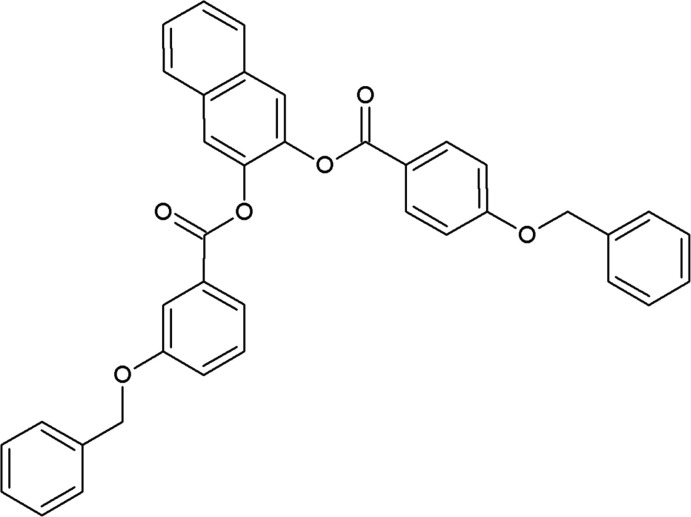


Our team is studying new bent-core liquid crystals with naphthalene rings as a rigid core (Srinivasa *et al.*, 2018 ▸) and, as part of that work, we have performed a simple coupling reaction between 1,2-di­hydroxy­naphthalene and 3-benz­yl­oxybenzoic acid to construct the title mol­ecule. It is a bent-type non-liquid crystal material, possibly due to the absence of alkyl chains/polar moiety at the ends of the mol­ecule.

## 2. Structural commentary

**Figure 1:**
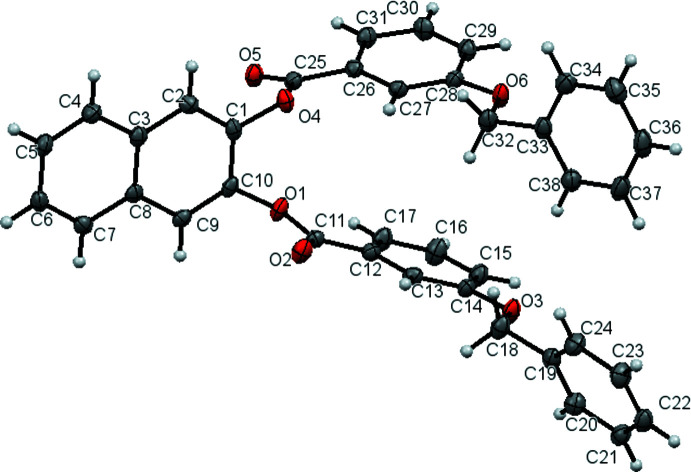
The mol­ecular structure of the title compound, showing displacement ellipsoids drawn at the 50% probability level.

The title compound crystallizes with one mol­ecule in the asym­metric unit (Fig. 1 ▸) in the space group *P*2_1_/*n*. The dihedral angles between the C1–C10 naphthalene ring system (r.m.s. deviation = 0.022 Å) and its pendant C26–C31 (*A*) and C12–C17 (*B*) benz­yloxy rings are 88.05 (7) and 80.84 (7)°, respectively. The dihedral angles between the *A* and *B* rings and their attached C33–C38 and C19–C24 phenyl rings are 49.15 (8) and 80.78 (8)°, respectively. Key torsion angles include C1—O4—C25—C26 [−160.98 (13)°], C28—O2—C32—C33 [−172.04 (14)°], C10—O1—C11—C12 [−168.94 (14)°] and C14—O3—C18—C19 [172.84 (14)°]. Otherwise, the geometrical data for the title compound may be regarded as normal.

## 3. Supra­molecular features

**Figure 2:**
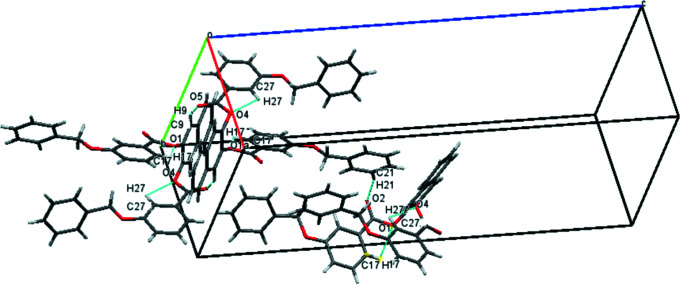
Partial packing diagram showing the C—H⋯O inter­actions.

In the crystal, the mol­ecules are linked by numerous C—H⋯O and C—H⋯π inter­actions (Table 1 ▸). Prominent packing features include a *C*(11) chain (arising from the C21—H21⋯O2^ii^ hydrogen bond), which runs along [010], and centrosymmetric 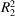 (12) loops (arising from the C9—H9⋯O5^i^ hydrogen bond) between the mol­ecules as shown in Fig. 2 ▸. These, and the C—H⋯π inter­actions, link the mol­ecules into a three-dimensional network (see Figs. S1 and S2 in the supporting information).

## 4. Hirshfeld surface analysis

**Figure 3:**
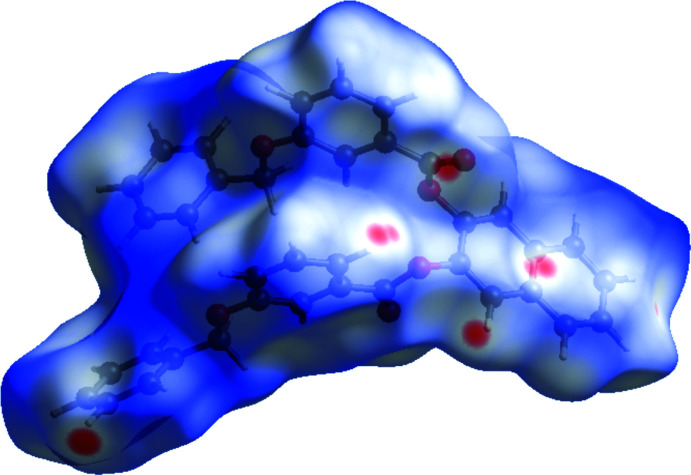
The Hirshfeld surface of the title compound mapped over *d*_norm_.

**Figure 4:**
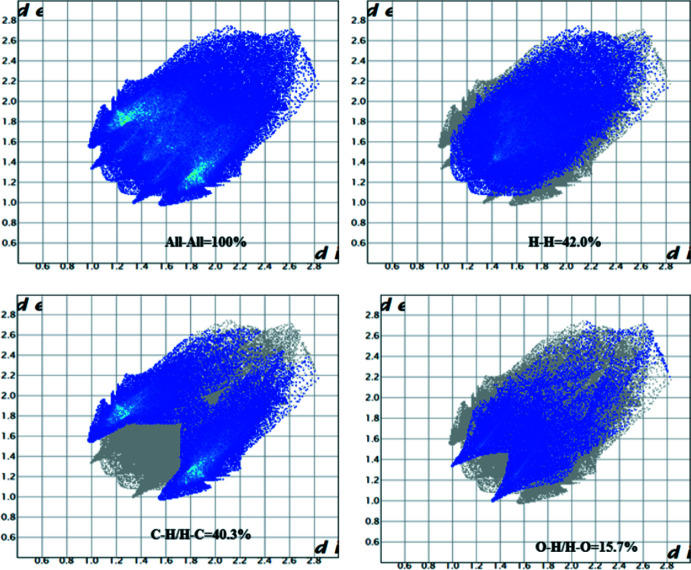
The 2D fingerprint plots for the title compound, showing C⋯H/H⋯C, H⋯H/H⋯H, O⋯H/H⋯O and O⋯O/O⋯O contacts.

**Figure 5:**
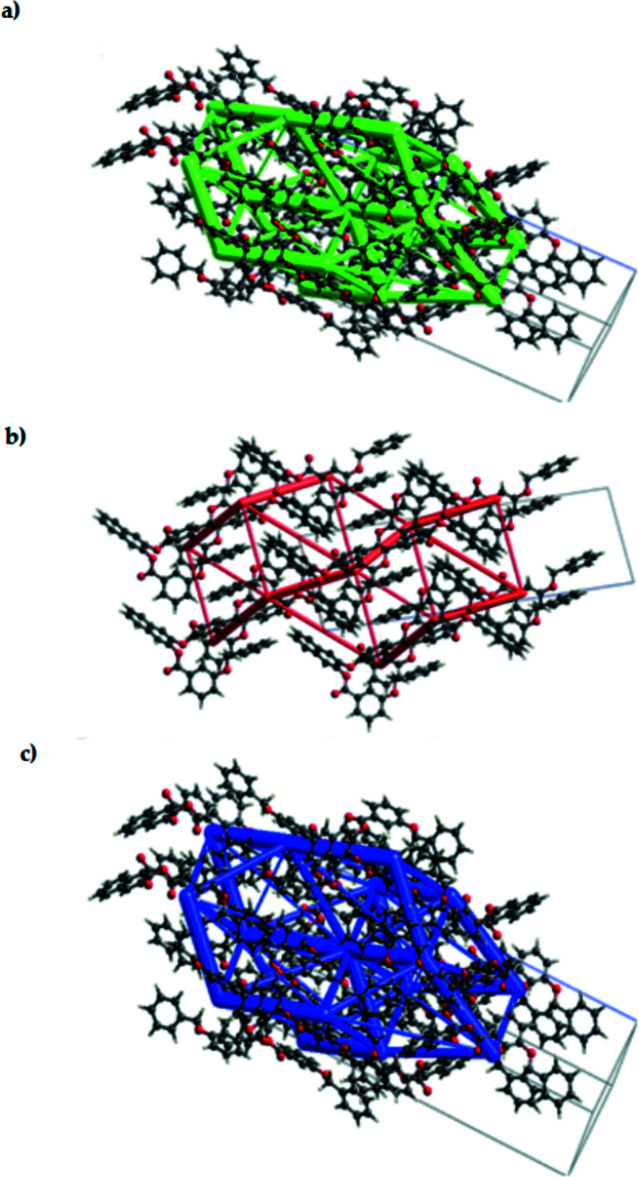
Energy frameworks calculated for the title compound, viewed along the *a*-axis direction, showing (*a*) Coulomb potential force, (*b*) dispersion force and (*c*) total energy diagrams. The cylindrical radii are proportional to the relative strength of the corresponding energies and they were adjusted to a cutoff value of 5 kJ mol^−1^.

The title mol­ecule was subjected to Hirshfeld surface analysis (Spackman & Jayatilaka, 2009 ▸) and the two-dimensional (2D) fingerprint plots (McKinnon *et al.*, 2007 ▸) were generated with *CrystalExplorer17* (Turner *et al.*, 2017 ▸). The Hirshfeld surface mapped on *d*_norm_ is shown in Fig. 3 ▸ and the overall 2D fingerprint plot and those delineated into H⋯H (42.3%), C⋯H/H⋯C (40.3%) and O⋯H/H⋯O (15.7%) contacts, together with their relative contributions to the Hirshfeld surface, are illustrated in Fig. 4 ▸. The inter­action energies for the title compound were calculated at the HF/3-21G quantum level of theory in *CrystalExplorer*. The four energy variables that make up the total inter­molecular inter­action energy (*E*_tot_) are electrostatic (*E*_ele_), polarization (*E*_pol_), dispersion (*E*_disp_) and exchange–repulsion (*E*_rep_), and the cylinder-shaped energy frameworks represent the relative strengths of the inter­action energies in individual directions, as well as the topologies of pairwise inter­molecular inter­action energies within the crystal (Mackenzie *et al.*, 2017 ▸). The energies between mol­ecular pairs are depicted as cylinders connecting the centroids of two mol­ecules, with the radius of the cylinder equal to the amount of inter­action energy between the mol­ecules (Wu *et al.*, 2020 ▸). The net inter­action energies for the title compound are *E*_ele_ = −56.3 kJ mol^−1^, *E*_pol_ = −30.4.0 kJ mol^−1^, *E*_dis_ = −428.6 kJ mol^−1^ and *E*_rep_ = 160.4 kJ mol^−1^, with a total inter­action energy *E*_tot_ of −333.3 kJ mol^−1^. Therefore, *E*_dis_ is the major inter­action energy in the title compound. The energy framework showing the electrostatic potential force, dispersion force and total energy diagrams are shown in Fig. 5 ▸. The cylindrical radii are proportional to the relative strength of the corresponding energies and they were adjusted to the same scale factor of 50 with a cutoff value of 5 kJ mol^−1^.

## 5. Database survey

A search of the Cambridge Structural Database (CSD; Version 5.43, update of March 2022: Groom *et al.*, 2016 ▸) for the naphthalene-2,3-diyl fragment gave 26 hits, of which six mol­ecules are similar to the title compound, with CSD refcodes WAFRII, WAFROO, WAFRUU, WAFSAB, WAFSEF and WAFSIJ (Rutherford *et al.*, 2020 ▸). There exist inter­molecular inter­actions dominated by π–π stacking and C—H⋯π inter­actions involving the arene rings in the benzoate fragments and the arene ring in the tetra­hydro­napthalene moiety. A ‘thermosalient phase transition effect’ was studied in the compounds coded QIBMUM and QIBMUM01–QIBMUM06 (Tamboli *et al.*, 2013 ▸), which feature a naphthalene-2,3-diyl bis­(4-fluoro­benzoate) fragment. The presence of π–π stacking and C—H⋯O and C—H⋯F inter­actions appear to play an important role in determining the mol­ecular conformations. The crystal structure analyses of the polymorphic structures coded DOPPAB, DOPPAB01, DOPPAB02, DOPPOP, DOPPOP01 and DOPQAC (Tamboli *et al.*, 2018 ▸) revealed weak inter­molecular inter­actions, such as C—H⋯O, C—H⋯π and π–π stacking, as also seen in the title mol­ecule. These inter­actions are actively involved in mol­ecular aggregation, which results in the polymorphic modifications, if they are subjected to thermal transformation. Here, all the mol­ecules crystallize in the space group *Pbcn* or *P*2/*c*. The crystal structure analyses of IJAGIJ01 to IJAGIJ05 (Tamboli *et al.*, 2014 ▸) are polymorphs of isomeric napthalene-2,3-diol ditoluates, in which the inter­molecular inter­actions, such as C—H⋯O, C—H⋯π and π–π stacking, are similar to the inter­actions present in the title mol­ecule.

## 6. Synthesis and crystalization

Under an inert atmosphere, 1,2-di­hydroxy­naphthalene (1.00 mmol), a catalytic amount of 4-di­methyl­amino­pyridine and 3-benzyl­oxybenzoic acid (2.00 mmol) were dissolved in 50 ml of dry di­chloro­methane (DCM). The above mixture was stirred for 2 h at room temperature with a solution of *N*,*N*-di­cyclo­hexyl­carbodi­imide (1.2 mmol) in DCM (20 ml). Filtration was used to remove the precipitated *N*,*N*-di­cyclo­hexyl­urea and the solvent was evaporated. To obtain the pure product, the solid residue was purified using column chromatography on silica gel with DCM as an eluent, followed by recrystallization from ethyl alcohol solution.

## 7. Refinement

Crystal data, data collection and structure refinement details are summarized in Table 2 ▸. H atoms were positioned geometrically (C—H = 0.93 Å) and refined as riding with *U*_iso_(H) = 1.2*U*_eq_(C).

## Supplementary Material

Crystal structure: contains datablock(s) I, global. DOI: 10.1107/S2056989023005571/hb8069sup1.cif

Structure factors: contains datablock(s) I. DOI: 10.1107/S2056989023005571/hb8069Isup4.hkl

Supplementary Figures showing C--H...pi interactions. DOI: 10.1107/S2056989023005571/hb8069sup3.docx

CCDC reference: 2271880

Additional supporting information:  crystallographic information; 3D view; checkCIF report

## Figures and Tables

**Table 1:** Hydrogen-bond geometry (Å, °) *Cg*1, *Cg*2, *Cg*4, *Cg*5, *Cg*6 and *Cg*7 are the centroids of the C1–C3/C8–C10, C3–C8, C19–C24, C26–C31, C33–C38 and C1–C10 rings, respectively.

*D*—H⋯*A*	*D*—H	H⋯*A*	*D*⋯*A*	*D*—H⋯*A*
C9—H9⋯O5^i^	0.93	2.52	3.258 (2)	136
C21—H21⋯O2^ii^	0.93	2.49	3.378 (2)	160
C4—H4⋯*Cg*5^iii^	0.93	2.60	3.4949 (19)	163
C16—H16⋯*Cg*2^i^	0.93	2.95	3.6955 (18)	139
C17—H17⋯*Cg*1^i^	0.93	2.90	3.7480 (17)	152
C17—H17⋯*Cg*7^i^	0.93	2.91	3.6342 (17)	135
C18—H18*A*⋯*Cg*6^ii^	0.97	2.66	3.5201 (18)	148
C30—H30⋯*Cg*1^iv^	0.93	2.90	3.7078 (17)	146
C31—H31⋯*Cg*2^iv^	0.93	2.69	3.5449 (17)	154
C31—H31⋯*Cg*7^iv^	0.93	2.95	3.6130 (16)	130
C32—H32*A*⋯*Cg*4^v^	0.97	2.82	3.5525 (18)	133
C15—H15⋯*Cg*6^vi^	0.93	2.97	3.6860 (18)	135

Symmetry codes: (i) 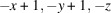 ; (ii) 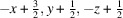 ; (iii) 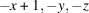 ; (iv) 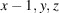 ; (v) 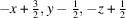 ; (vi) 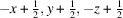 .

**Table 2:** Experimental details

Crystal data	
Chemical formula	C_38_H_28_O_6_
*M*_r_	580.60
Crystal system, space group	Monoclinic, *P*2_1_/*n*
Temperature (K)	302
*a*, *b*, *c* (Å)	9.5219 (2), 10.1010 (2), 30.7050 (8)
β (°)	96.666 (1)
*V* (Å^3^)	2933.26 (11)
*Z*	4
Radiation type	Cu *K*α
μ (mm^−1^)	0.72
Crystal size (mm)	0.32 × 0.28 × 0.21

Data collection	
Diffractometer	Bruker SMART APEXII CCD
No. of measured, independent and observed [*I* > 2σ(*I*)] reflections	14741, 4775, 4298
*R*_int_	0.032
(sin θ/λ)_max_ (Å^−1^)	0.585

Refinement	
*R*[*F* ^2^ > 2σ(*F* ^2^)], *wR*(*F* ^2^), *S*	0.042, 0.142, 1.06
No. of reflections	4775
No. of parameters	397
H-atom treatment	H-atom parameters constrained
Δρ_max_, Δρ_min_ (e Å^−3^)	0.24, −0.23

Computer programs: *APEX3* and *SAINT* (Bruker, 2014 ▸), *SHELXT* (Sheldrick, 2015*a* ▸), *SHELXL* (Sheldrick, 2015*b* ▸) and *Mercury* (Macrae *et al.*, 2020 ▸).

## supplementary crystallographic information

### Crystal data

**Table:**

C_38_H_28_O_6_	Prism
*M**_r_* = 580.60	*D*_x_ = 1.315 Mg m^−^^3^
Monoclinic, *P*2_1_/*n*	Melting point: 417 K
Hall symbol: -P 2yn	Cu *K*α radiation, λ = 1.54178 Å
*a* = 9.5219 (2) Å	Cell parameters from 4775 reflections
*b* = 10.1010 (2) Å	θ = 0.3–25°
*c* = 30.7050 (8) Å	µ = 0.72 mm^−^^1^
β = 96.666 (1)°	*T* = 302 K
*V* = 2933.26 (11) Å^3^	Rod, colourless
*Z* = 4	0.32 × 0.28 × 0.21 mm
*F*(000) = 1216	

### Data collection

**Table:**

Bruker SMART APEXII CCD diffractometer	4298 reflections with *I* > 2σ(*I*)
Radiation source: fine-focus sealed tube	*R*_int_ = 0.032
Graphite monochromator	θ_max_ = 64.5°, θ_min_ = 4.6°
Detector resolution: 2.06 pixels mm^-1^	*h* = −11→10
ω scans	*k* = −11→7
14741 measured reflections	*l* = −32→35
4775 independent reflections	

### Refinement

**Table:**

Refinement on *F*^2^	Primary atom site location: structure-invariant direct methods
Least-squares matrix: full	Secondary atom site location: difference Fourier map
*R*[*F*^2^ > 2σ(*F*^2^)] = 0.042	Hydrogen site location: inferred from neighbouring sites
*wR*(*F*^2^) = 0.142	H-atom parameters constrained
*S* = 1.06	*w* = 1/[σ^2^(*F*_o_^2^) + (0.0997*P*)^2^ + 0.5664*P*] where *P* = (*F*_o_^2^ + 2*F*_c_^2^)/3
4775 reflections	(Δ/σ)_max_ < 0.001
397 parameters	Δρ_max_ = 0.24 e Å^−^^3^
0 restraints	Δρ_min_ = −0.23 e Å^−^^3^
1 constraint	

### Special details

**Table:**

Geometry. All esds (except the esd in the dihedral angle between two l.s. planes) are estimated using the full covariance matrix. The cell esds are taken into account individually in the estimation of esds in distances, angles and torsion angles; correlations between esds in cell parameters are only used when they are defined by crystal symmetry. An approximate (isotropic) treatment of cell esds is used for estimating esds involving l.s. planes.

### Fractional atomic coordinates and isotropic or equivalent isotropic displacement parameters (Å^2^)

**Table:**

	*x*	*y*	*z*	*U*_iso_*/*U*_eq_	
O1	0.58939 (11)	0.43382 (12)	0.05018 (3)	0.0232 (3)	
O2	0.77634 (11)	0.38287 (13)	0.09926 (3)	0.0272 (3)	
O3	0.58453 (11)	0.47609 (13)	0.24604 (3)	0.0268 (3)	
O4	0.50928 (11)	0.18476 (12)	0.02981 (3)	0.0225 (3)	
O5	0.34497 (11)	0.27300 (12)	−0.02087 (3)	0.0248 (3)	
O6	0.25879 (11)	0.13423 (13)	0.16475 (3)	0.0253 (3)	
C1	0.61513 (15)	0.23968 (17)	0.00711 (5)	0.0201 (4)	
C2	0.67835 (16)	0.16830 (17)	−0.02259 (5)	0.0213 (4)	
H2	0.649084	0.082186	−0.029440	0.026*	
C3	0.78971 (15)	0.22628 (17)	−0.04315 (5)	0.0204 (4)	
C4	0.86373 (17)	0.15418 (18)	−0.07275 (5)	0.0243 (4)	
H4	0.838208	0.067021	−0.079451	0.029*	
C5	0.97232 (17)	0.21079 (18)	−0.09165 (5)	0.0254 (4)	
H5	1.021228	0.161481	−0.110563	0.031*	
C6	1.01010 (16)	0.34325 (18)	−0.08256 (5)	0.0237 (4)	
H6	1.083712	0.381211	−0.095640	0.028*	
C7	0.93960 (15)	0.41691 (18)	−0.05465 (5)	0.0212 (4)	
H7	0.963984	0.505156	−0.049498	0.025*	
C8	0.82939 (15)	0.35947 (17)	−0.03349 (4)	0.0187 (3)	
C9	0.76033 (15)	0.43085 (17)	−0.00204 (5)	0.0194 (3)	
H9	0.785118	0.518167	0.004687	0.023*	
C10	0.65814 (15)	0.37016 (17)	0.01796 (4)	0.0200 (4)	
C11	0.65782 (15)	0.42547 (17)	0.09173 (5)	0.0199 (4)	
C12	0.56915 (16)	0.47090 (16)	0.12536 (5)	0.0203 (4)	
C13	0.62360 (16)	0.45161 (17)	0.16931 (5)	0.0213 (4)	
H13	0.712355	0.413805	0.176414	0.026*	
C14	0.54323 (16)	0.48970 (17)	0.20201 (5)	0.0216 (4)	
C15	0.40898 (17)	0.54473 (18)	0.19114 (5)	0.0266 (4)	
H15	0.354379	0.568536	0.213113	0.032*	
C16	0.35781 (17)	0.5636 (2)	0.14761 (5)	0.0307 (4)	
H16	0.269350	0.601978	0.140482	0.037*	
C17	0.43662 (17)	0.52605 (19)	0.11437 (5)	0.0272 (4)	
H17	0.400928	0.537764	0.085129	0.033*	
C18	0.72408 (17)	0.42395 (19)	0.25874 (5)	0.0265 (4)	
H18A	0.793380	0.474299	0.244862	0.032*	
H18B	0.728805	0.332338	0.249542	0.032*	
C19	0.75487 (16)	0.43351 (18)	0.30777 (5)	0.0234 (4)	
C20	0.73388 (16)	0.55202 (18)	0.32920 (5)	0.0250 (4)	
H20	0.696094	0.624617	0.313295	0.030*	
C21	0.76914 (16)	0.56235 (19)	0.37426 (5)	0.0262 (4)	
H21	0.754780	0.641792	0.388404	0.031*	
C22	0.82545 (17)	0.45503 (19)	0.39814 (5)	0.0282 (4)	
H22	0.849553	0.462230	0.428278	0.034*	
C23	0.84571 (19)	0.3374 (2)	0.37712 (5)	0.0316 (4)	
H23	0.883045	0.264814	0.393102	0.038*	
C24	0.81042 (18)	0.32703 (19)	0.33198 (5)	0.0286 (4)	
H24	0.824505	0.247257	0.318000	0.034*	
C25	0.37343 (15)	0.22511 (16)	0.01482 (5)	0.0192 (3)	
C26	0.27380 (16)	0.20240 (16)	0.04756 (5)	0.0194 (3)	
C27	0.32213 (16)	0.17695 (16)	0.09144 (5)	0.0199 (3)	
H27	0.418421	0.168975	0.100463	0.024*	
C28	0.22447 (16)	0.16371 (17)	0.12146 (5)	0.0204 (4)	
C29	0.08046 (16)	0.17887 (18)	0.10773 (5)	0.0235 (4)	
H29	0.015499	0.173477	0.128056	0.028*	
C30	0.03403 (16)	0.20179 (18)	0.06419 (5)	0.0250 (4)	
H30	−0.062326	0.209607	0.055252	0.030*	
C31	0.12965 (16)	0.21333 (17)	0.03353 (5)	0.0233 (4)	
H31	0.098083	0.228097	0.004124	0.028*	
C32	0.40533 (16)	0.1048 (2)	0.17832 (5)	0.0267 (4)	
H32A	0.462305	0.183689	0.176271	0.032*	
H32B	0.438343	0.037409	0.159440	0.032*	
C33	0.41890 (16)	0.05652 (19)	0.22477 (5)	0.0247 (4)	
C34	0.39745 (18)	−0.0760 (2)	0.23388 (5)	0.0311 (4)	
H34	0.369988	−0.134322	0.211041	0.037*	
C35	0.41657 (19)	−0.1225 (2)	0.27679 (6)	0.0350 (4)	
H35	0.401233	−0.211366	0.282630	0.042*	
C36	0.45865 (18)	−0.0357 (2)	0.31084 (5)	0.0331 (4)	
H36	0.473278	−0.066367	0.339563	0.040*	
C37	0.47870 (18)	0.0964 (2)	0.30192 (5)	0.0330 (4)	
H37	0.506345	0.154630	0.324773	0.040*	
C38	0.45797 (17)	0.1431 (2)	0.25914 (5)	0.0281 (4)	
H38	0.470290	0.232544	0.253504	0.034*	

### Atomic displacement parameters (Å^2^)

**Table:**

	*U*^11^	*U*^22^	*U*^33^	*U*^12^	*U*^13^	*U*^23^
O1	0.0207 (5)	0.0346 (7)	0.0141 (5)	0.0060 (5)	0.0012 (4)	−0.0030 (4)
O2	0.0200 (6)	0.0404 (8)	0.0207 (6)	0.0065 (5)	−0.0001 (4)	−0.0027 (5)
O3	0.0203 (6)	0.0440 (8)	0.0159 (5)	0.0045 (5)	0.0003 (4)	−0.0018 (5)
O4	0.0168 (5)	0.0302 (7)	0.0212 (5)	0.0025 (4)	0.0048 (4)	0.0067 (5)
O5	0.0230 (6)	0.0345 (7)	0.0167 (6)	0.0007 (5)	0.0013 (4)	0.0025 (5)
O6	0.0190 (6)	0.0417 (8)	0.0151 (5)	0.0018 (5)	0.0023 (4)	0.0017 (5)
C1	0.0150 (7)	0.0286 (10)	0.0167 (7)	0.0005 (6)	0.0015 (5)	0.0053 (6)
C2	0.0201 (8)	0.0218 (9)	0.0217 (8)	−0.0004 (6)	0.0013 (6)	0.0019 (6)
C3	0.0181 (7)	0.0276 (10)	0.0151 (7)	0.0010 (6)	−0.0002 (6)	0.0012 (6)
C4	0.0255 (8)	0.0243 (10)	0.0233 (8)	−0.0003 (7)	0.0036 (6)	−0.0029 (7)
C5	0.0225 (8)	0.0342 (11)	0.0203 (8)	0.0023 (7)	0.0050 (6)	−0.0025 (7)
C6	0.0182 (7)	0.0352 (11)	0.0176 (7)	−0.0031 (7)	0.0021 (6)	0.0023 (7)
C7	0.0198 (8)	0.0247 (9)	0.0179 (7)	−0.0027 (6)	−0.0025 (6)	0.0027 (6)
C8	0.0165 (7)	0.0250 (9)	0.0135 (7)	−0.0003 (6)	−0.0025 (5)	0.0008 (6)
C9	0.0184 (7)	0.0223 (9)	0.0163 (7)	0.0014 (6)	−0.0023 (6)	−0.0015 (6)
C10	0.0172 (7)	0.0293 (10)	0.0127 (7)	0.0045 (6)	−0.0011 (5)	−0.0018 (6)
C11	0.0191 (8)	0.0226 (9)	0.0174 (7)	−0.0012 (6)	−0.0003 (6)	−0.0003 (6)
C12	0.0196 (8)	0.0224 (9)	0.0187 (7)	−0.0014 (6)	0.0014 (6)	−0.0019 (6)
C13	0.0170 (7)	0.0244 (9)	0.0222 (8)	−0.0001 (6)	0.0005 (6)	−0.0021 (6)
C14	0.0221 (8)	0.0272 (9)	0.0154 (7)	−0.0031 (7)	0.0010 (6)	−0.0021 (6)
C15	0.0213 (8)	0.0364 (11)	0.0226 (8)	0.0026 (7)	0.0041 (6)	−0.0051 (7)
C16	0.0202 (8)	0.0458 (12)	0.0256 (8)	0.0096 (7)	0.0008 (6)	−0.0015 (7)
C17	0.0243 (8)	0.0377 (11)	0.0185 (7)	0.0042 (7)	−0.0015 (6)	−0.0001 (7)
C18	0.0207 (8)	0.0391 (11)	0.0197 (8)	0.0048 (7)	0.0015 (6)	−0.0025 (7)
C19	0.0159 (7)	0.0341 (10)	0.0200 (8)	−0.0002 (7)	0.0018 (6)	−0.0012 (7)
C20	0.0219 (8)	0.0292 (10)	0.0236 (8)	0.0039 (7)	0.0013 (6)	0.0014 (7)
C21	0.0205 (8)	0.0339 (11)	0.0244 (8)	0.0000 (7)	0.0035 (6)	−0.0071 (7)
C22	0.0235 (8)	0.0430 (12)	0.0180 (8)	−0.0026 (7)	0.0015 (6)	−0.0003 (7)
C23	0.0358 (10)	0.0349 (11)	0.0235 (8)	0.0033 (8)	0.0011 (7)	0.0057 (7)
C24	0.0312 (9)	0.0290 (10)	0.0258 (8)	0.0025 (7)	0.0046 (7)	−0.0029 (7)
C25	0.0188 (7)	0.0207 (9)	0.0178 (7)	−0.0004 (6)	0.0013 (6)	−0.0023 (6)
C26	0.0193 (8)	0.0200 (9)	0.0190 (8)	0.0004 (6)	0.0027 (6)	−0.0011 (6)
C27	0.0160 (7)	0.0235 (9)	0.0199 (8)	−0.0003 (6)	0.0010 (6)	−0.0016 (6)
C28	0.0222 (8)	0.0231 (9)	0.0157 (7)	−0.0009 (6)	0.0017 (6)	−0.0018 (6)
C29	0.0187 (8)	0.0291 (10)	0.0234 (8)	0.0019 (6)	0.0054 (6)	−0.0003 (7)
C30	0.0156 (7)	0.0333 (10)	0.0258 (8)	0.0033 (7)	0.0017 (6)	0.0023 (7)
C31	0.0217 (8)	0.0277 (10)	0.0198 (7)	0.0013 (7)	−0.0004 (6)	0.0019 (6)
C32	0.0186 (8)	0.0416 (11)	0.0195 (8)	−0.0022 (7)	0.0006 (6)	0.0021 (7)
C33	0.0176 (7)	0.0379 (11)	0.0188 (8)	−0.0019 (7)	0.0025 (6)	0.0009 (7)
C34	0.0296 (9)	0.0386 (12)	0.0250 (9)	−0.0057 (8)	0.0032 (7)	−0.0028 (7)
C35	0.0330 (9)	0.0406 (12)	0.0319 (9)	−0.0028 (8)	0.0064 (7)	0.0098 (8)
C36	0.0279 (9)	0.0519 (13)	0.0195 (8)	0.0004 (8)	0.0030 (6)	0.0083 (8)
C37	0.0308 (9)	0.0491 (13)	0.0189 (8)	−0.0036 (8)	0.0021 (7)	−0.0025 (8)
C38	0.0247 (8)	0.0362 (11)	0.0235 (8)	−0.0024 (7)	0.0034 (6)	0.0006 (7)

### Geometric parameters (Å, º)

**Table:**

O1—C11	1.3662 (18)	C18—C19	1.503 (2)
O1—C10	1.4040 (18)	C18—H18A	0.9700
O2—C11	1.2049 (19)	C18—H18B	0.9700
O3—C14	1.3702 (18)	C19—C24	1.378 (3)
O3—C18	1.4404 (19)	C19—C20	1.392 (2)
O4—C25	1.3831 (18)	C20—C21	1.389 (2)
O4—C1	1.4045 (18)	C20—H20	0.9300
O5—C25	1.1998 (18)	C21—C22	1.382 (3)
O6—C28	1.3642 (18)	C21—H21	0.9300
O6—C32	1.4401 (18)	C22—C23	1.376 (3)
C1—C2	1.357 (2)	C22—H22	0.9300
C1—C10	1.409 (2)	C23—C24	1.392 (2)
C2—C3	1.422 (2)	C23—H23	0.9300
C2—H2	0.9300	C24—H24	0.9300
C3—C4	1.415 (2)	C25—C26	1.478 (2)
C3—C8	1.419 (2)	C26—C31	1.394 (2)
C4—C5	1.368 (2)	C26—C27	1.396 (2)
C4—H4	0.9300	C27—C28	1.390 (2)
C5—C6	1.405 (3)	C27—H27	0.9300
C5—H5	0.9300	C28—C29	1.395 (2)
C6—C7	1.368 (2)	C29—C30	1.378 (2)
C6—H6	0.9300	C29—H29	0.9300
C7—C8	1.421 (2)	C30—C31	1.388 (2)
C7—H7	0.9300	C30—H30	0.9300
C8—C9	1.425 (2)	C31—H31	0.9300
C9—C10	1.356 (2)	C32—C33	1.498 (2)
C9—H9	0.9300	C33—C34	1.387 (3)
C11—C12	1.480 (2)	C33—C38	1.388 (2)
C12—C17	1.385 (2)	C34—C35	1.391 (2)
C12—C13	1.402 (2)	C34—H34	0.9300
C13—C14	1.386 (2)	C35—C36	1.388 (3)
C13—H13	0.9300	C35—H35	0.9300
C14—C15	1.398 (2)	C36—C37	1.379 (3)
C15—C16	1.382 (2)	C36—H36	0.9300
C15—H15	0.9300	C37—C38	1.388 (2)
C16—C17	1.388 (2)	C37—H37	0.9300
C16—H16	0.9300	C38—H38	0.9300
C17—H17	0.9300		
			
C11—O1—C10	114.77 (11)	H18A—C18—H18B	108.4
C14—O3—C18	116.99 (12)	C24—C19—C20	118.91 (14)
C25—O4—C1	114.56 (11)	C24—C19—C18	120.55 (16)
C28—O6—C32	116.20 (11)	C20—C19—C18	120.46 (15)
C2—C1—C10	121.11 (14)	C21—C20—C19	120.28 (16)
C2—C1—O4	121.57 (15)	C21—C20—H20	119.9
C10—C1—O4	117.25 (13)	C19—C20—H20	119.9
C2—C1—O4	121.57 (15)	C22—C21—C20	120.24 (17)
C10—C1—O4	117.25 (13)	C22—C21—H21	119.9
C1—C2—C3	119.43 (15)	C20—C21—H21	119.9
C1—C2—H2	120.3	C21—C22—C23	119.68 (15)
C3—C2—H2	120.3	C21—C22—H22	120.2
C4—C3—C2	121.73 (15)	C23—C22—H22	120.2
C4—C3—C8	118.80 (14)	C22—C23—C24	120.11 (17)
C2—C3—C8	119.46 (14)	C22—C23—H23	119.9
C5—C4—C3	120.97 (16)	C24—C23—H23	119.9
C5—C4—H4	119.5	C19—C24—C23	120.77 (17)
C3—C4—H4	119.5	C19—C24—H24	119.6
C4—C5—C6	120.16 (15)	C23—C24—H24	119.6
C4—C5—H5	119.9	O5—C25—O4	121.75 (13)
C6—C5—H5	119.9	O5—C25—O4	121.75 (13)
C5—C6—C7	120.62 (15)	O5—C25—C26	126.09 (14)
C5—C6—H6	119.7	O4—C25—C26	112.15 (12)
C7—C6—H6	119.7	O4—C25—C26	112.15 (12)
C6—C7—C8	120.43 (16)	C31—C26—C27	121.00 (14)
C6—C7—H7	119.8	C31—C26—C25	117.65 (14)
C8—C7—H7	119.8	C27—C26—C25	121.28 (13)
C7—C8—C9	121.77 (15)	C28—C27—C26	119.14 (14)
C7—C8—C3	118.97 (14)	C28—C27—H27	120.4
C9—C8—C3	119.24 (14)	C26—C27—H27	120.4
C10—C9—C8	119.32 (15)	O6—C28—C27	124.34 (13)
C10—C9—H9	120.3	O6—C28—C29	115.74 (13)
C8—C9—H9	120.3	C27—C28—C29	119.91 (14)
C9—C10—O1	121.99 (15)	C30—C29—C28	120.33 (14)
C9—C10—O1	121.99 (15)	C30—C29—H29	119.8
C9—C10—C1	121.34 (14)	C28—C29—H29	119.8
O1—C10—C1	116.66 (13)	C29—C30—C31	120.67 (14)
O1—C10—C1	116.66 (13)	C29—C30—H30	119.7
O2—C11—O1	122.40 (14)	C31—C30—H30	119.7
O2—C11—O1	122.40 (14)	C26—C31—C30	118.91 (14)
O2—C11—C12	125.04 (13)	C26—C31—H31	120.5
O1—C11—C12	112.55 (12)	C30—C31—H31	120.5
O1—C11—C12	112.55 (12)	O6—C32—C33	108.43 (12)
C17—C12—C13	121.00 (14)	O6—C32—H32A	110.0
C17—C12—C11	122.14 (13)	C33—C32—H32A	110.0
C13—C12—C11	116.82 (13)	O6—C32—H32B	110.0
C14—C13—C12	119.04 (14)	C33—C32—H32B	110.0
C14—C13—H13	120.5	H32A—C32—H32B	108.4
C12—C13—H13	120.5	C34—C33—C38	119.16 (15)
O3—C14—C13	124.59 (14)	C34—C33—C32	120.50 (16)
O3—C14—C15	115.16 (13)	C38—C33—C32	120.30 (17)
C13—C14—C15	120.25 (14)	C33—C34—C35	120.74 (17)
C16—C15—C14	119.72 (15)	C33—C34—H34	119.6
C16—C15—H15	120.1	C35—C34—H34	119.6
C14—C15—H15	120.1	C36—C35—C34	119.64 (19)
C15—C16—C17	120.90 (15)	C36—C35—H35	120.2
C15—C16—H16	119.5	C34—C35—H35	120.2
C17—C16—H16	119.5	C35—C36—C37	119.75 (16)
C12—C17—C16	119.08 (14)	C35—C36—H36	120.1
C12—C17—H17	120.5	C37—C36—H36	120.1
C16—C17—H17	120.5	C38—C37—C36	120.58 (17)
O3—C18—C19	108.31 (12)	C38—C37—H37	119.7
O3—C18—H18A	110.0	C36—C37—H37	119.7
C19—C18—H18A	110.0	C37—C38—C33	120.11 (19)
O3—C18—H18B	110.0	C37—C38—H38	119.9
C19—C18—H18B	110.0	C33—C38—H38	119.9
			
C25—O4—C1—C2	−103.88 (16)	C13—C14—C15—C16	−1.3 (3)
C25—O4—C1—C10	79.14 (16)	C14—C15—C16—C17	1.4 (3)
O4—C1—C2—C3	−176.96 (12)	C13—C12—C17—C16	0.6 (3)
O4—C1—C2—C3	−176.96 (12)	C11—C12—C17—C16	178.47 (16)
C1—C2—C3—C4	176.92 (14)	C15—C16—C17—C12	−1.0 (3)
C1—C2—C3—C8	−2.3 (2)	C14—O3—C18—C19	172.84 (14)
C2—C3—C4—C5	−178.84 (14)	O3—C18—C19—C24	132.87 (16)
C3—C4—C5—C6	−1.4 (2)	O3—C18—C19—C20	−50.1 (2)
C5—C6—C7—C8	1.7 (2)	C24—C19—C20—C21	0.3 (2)
C6—C7—C8—C9	175.77 (13)	C18—C19—C20—C21	−176.77 (14)
C6—C7—C8—C3	−2.6 (2)	C19—C20—C21—C22	0.1 (2)
C4—C3—C8—C7	1.5 (2)	C20—C21—C22—C23	−0.4 (2)
C2—C3—C8—C7	−179.20 (13)	C21—C22—C23—C24	0.4 (3)
C4—C3—C8—C9	−176.86 (13)	C20—C19—C24—C23	−0.3 (2)
C2—C3—C8—C9	2.4 (2)	C18—C19—C24—C23	176.77 (15)
C7—C8—C9—C10	−178.40 (13)	C22—C23—C24—C19	−0.1 (3)
C8—C9—C10—O1	177.98 (12)	C1—O4—C25—O5	18.8 (2)
C8—C9—C10—O1	177.98 (12)	C1—O4—C25—O4	0 (22)
C8—C9—C10—C1	−2.4 (2)	C1—O4—C25—C26	−160.98 (13)
C11—O1—C10—C9	−85.49 (17)	O5—C25—C26—C31	13.0 (3)
C11—O1—C10—C1	94.88 (15)	O4—C25—C26—C31	−167.24 (14)
C2—C1—C10—C9	2.5 (2)	O4—C25—C26—C31	−167.24 (14)
O4—C1—C10—C9	179.53 (13)	O5—C25—C26—C27	−164.05 (16)
O4—C1—C10—C9	179.53 (13)	O4—C25—C26—C27	15.7 (2)
C2—C1—C10—O1	−177.83 (13)	O4—C25—C26—C27	15.7 (2)
O4—C1—C10—O1	−0.83 (18)	C31—C26—C27—C28	−0.6 (2)
O4—C1—C10—O1	−0.83 (18)	C25—C26—C27—C28	176.37 (15)
C2—C1—C10—O1	−177.83 (13)	C32—O6—C28—C27	−5.3 (2)
O4—C1—C10—O1	−0.83 (18)	C32—O6—C28—C29	173.76 (15)
O4—C1—C10—O1	−0.83 (18)	C26—C27—C28—O6	177.57 (15)
C10—O1—C11—O2	9.7 (2)	C26—C27—C28—C29	−1.5 (3)
C10—O1—C11—C12	−168.94 (14)	O6—C28—C29—C30	−176.56 (15)
O2—C11—C12—C17	176.16 (17)	C27—C28—C29—C30	2.6 (3)
O1—C11—C12—C17	−5.3 (2)	C28—C29—C30—C31	−1.6 (3)
O1—C11—C12—C17	−5.3 (2)	C27—C26—C31—C30	1.6 (3)
O2—C11—C12—C13	−5.9 (3)	C25—C26—C31—C30	−175.49 (15)
O1—C11—C12—C13	172.69 (14)	C29—C30—C31—C26	−0.5 (3)
O1—C11—C12—C13	172.69 (14)	C28—O6—C32—C33	−172.04 (14)
C17—C12—C13—C14	−0.6 (3)	O6—C32—C33—C34	85.55 (19)
C11—C12—C13—C14	−178.55 (15)	O6—C32—C33—C38	−96.72 (18)
C18—O3—C14—C13	2.9 (2)	C32—C33—C34—C35	176.88 (15)
C18—O3—C14—C15	−177.96 (15)	C34—C35—C36—C37	1.1 (3)
C12—C13—C14—O3	179.99 (15)	C36—C37—C38—C33	−1.0 (3)
C12—C13—C14—C15	0.9 (2)	C34—C33—C38—C37	1.6 (2)
O3—C14—C15—C16	179.51 (16)	C32—C33—C38—C37	−176.12 (15)

### Hydrogen-bond geometry (Å, º)

**Table:**

*D*—H···*A*	*D*—H	H···*A*	*D*···*A*	*D*—H···*A*
C9—H9···O5^i^	0.93	2.52	3.258 (2)	136
C21—H21···O2^ii^	0.93	2.49	3.378 (2)	160
C4—H4···*Cg*5^iii^	0.93	2.60	3.4949 (19)	163
C16—H16···*Cg*2^i^	0.93	2.95	3.6955 (18)	139
C17—H17···*Cg*1^i^	0.93	2.90	3.7480 (17)	152
C17—H17···*Cg*7^i^	0.93	2.91	3.6342 (17)	135
C18—H18*A*···*Cg*6^ii^	0.97	2.66	3.5201 (18)	148
C30—H30···*Cg*1^iv^	0.93	2.90	3.7078 (17)	146
C31—H31···*Cg*2^iv^	0.93	2.69	3.5449 (17)	154
C31—H31···*Cg*7^iv^	0.93	2.95	3.6130 (16)	130
C32—H32*A*···*Cg*4^v^	0.97	2.82	3.5525 (18)	133
C15—H15···*Cg*6^vi^	0.93	2.97	3.6860 (18)	135

Symmetry codes: (i) −*x*+1, −*y*+1, −*z*; (ii) −*x*+3/2, *y*+1/2, −*z*+1/2; (iii) −*x*+1, −*y*, −*z*; (iv) *x*−1, *y*, *z*; (v) −*x*+3/2, *y*−1/2, −*z*+1/2; (vi) −*x*+1/2, *y*+1/2, −*z*+1/2.
