# Supplementary material for: Synthesis, crystal structure and Hirshfeld surface analysis of naphthalene-2,3-diyl bis­(3-benz­yl­oxy)benzoate
Source: Acta Crystallogr E Crystallogr Commun. 2023 Jul 4;79(Pt 8):686–9. doi: 10.1107/S2056989023005571 (PMC10439419; doi:10.1107/S2056989023005571)
Supplement: Supplementary file 3 [file e-79-00686-sup3.docx]

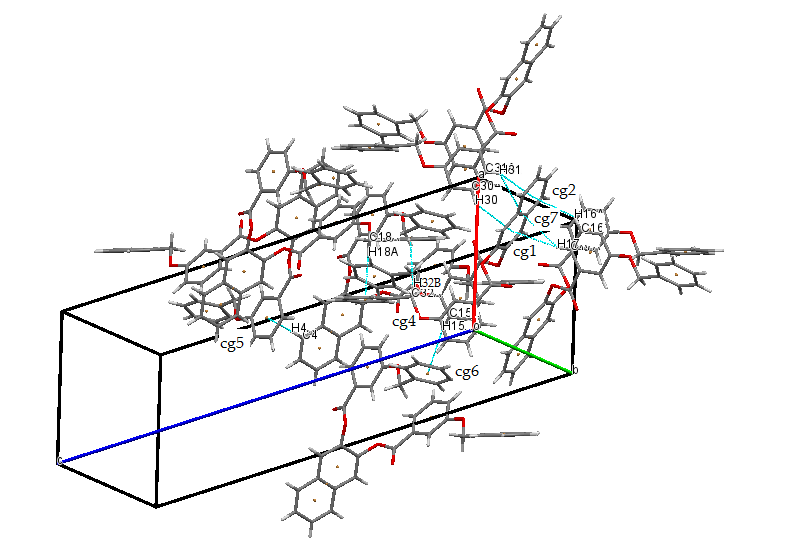


Figure S1: C—H⋅⋅⋅π interactions in the title compound (see Table 1 for ring designations).


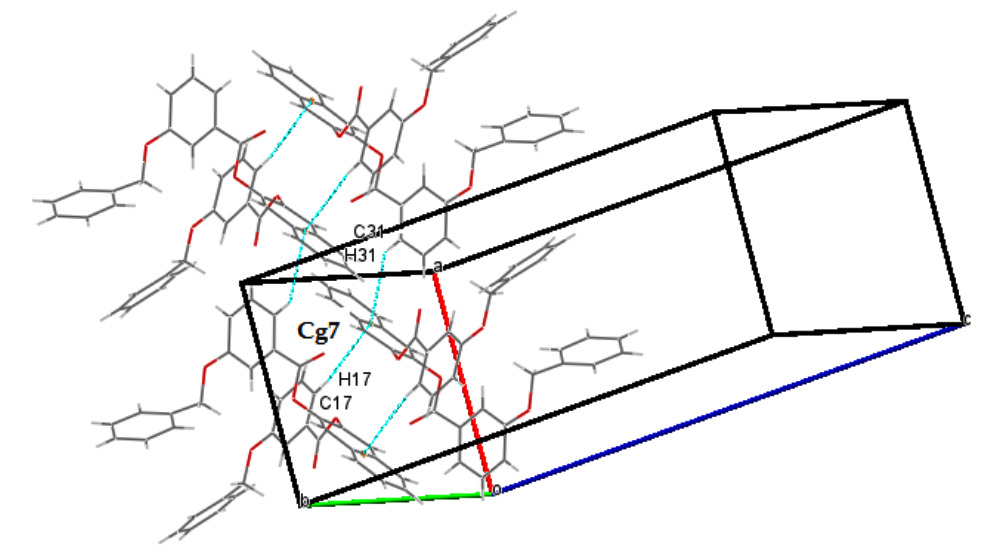


Figure S2: C—H⋅⋅⋅π interactions in the title compound involving the naphthalene ring.
